# Structure-Activity Relationships of Benzbromarone Metabolites and Derivatives as EYA Inhibitory Anti-Angiogenic Agents

**DOI:** 10.1371/journal.pone.0084582

**Published:** 2013-12-18

**Authors:** Ram Naresh Pandey, Tim Sen Wang, Emmanuel Tadjuidje, Matthew G. McDonald, Allan E. Rettie, Rashmi S. Hegde

**Affiliations:** 1 Division of Developmental Biology, Cincinnati Children’s Hospital Medical Center, Cincinnati, Ohio, United States of America; 2 Department of Medicinal Chemistry, University of Washington, Seattle, Washington, United States of America; UAE University, Faculty of Medicine & Health Sciences, United Arab Emirates

## Abstract

The tyrosine phosphatase activity of the phosphatase-transactivator protein Eyes Absent (EYA) is angiogenic through its roles in endothelial cell migration and tube formation. Benzbromarone, a known anti-gout agent, was previously identified as an inhibitor of EYA with anti-angiogenic properties. Here we show that the major metabolite of BBR, 6-hydroxy benzbromarone, is a significantly more potent inhibitor of cell migration, tubulogenesis and angiogenic sprouting. In contrast, other postulated metabolites of BBR such as 5-hydroxy benzbromaorne and 1’-hydroxy benzbromarone are less potent inhibitors of EYA tyrosine phosphatase activity as well as being less effective in cellular assays for endothelial cell migration and angiogenesis. Longer substituents at the 2 position of the benzofuran ring promoted EYA3 binding and inhibition, but were less effective in cellular assays, likely reflecting non-specific protein binding and a resulting reduction in free, bio-available inhibitor. The observed potency of 6-hydroxy benzbromarone is relevant in the context of the potential re-purposing of benzbromarone and its derivatives as anti-angiogenic agents. 6-hydroxy benzbromarone represents a metabolite with a longer half-life and greater pharmacological potency than the parent compound, suggesting that biotransformation of benzbromarone could contribute to its therapeutic activity.

## Introduction

The Eyes Absent (EYA) proteins are an unusual family of protein tyrosine phosphatases (PTP) originally described as part of a conserved pathway involved in cell-fate determination. In addition to the tyrosine phosphatase domain [1-3], they have a separate threonine phosphatase domain [4] and can act as transcriptional activators in complex with a DNA-binding partner, typically the SIX proteins [5]. These multi-functional proteins have been associated with many human disease states – loss of function being encountered in developmental disorders, and either over-expression or silencing being associated with many types of cancer (recently reviewed in [6]). High levels of EYA correlate with a worse outcome in malignant peripheral nerve sheath tumors (EYA4) [7], breast and ovarian cancers (EYA2) [8,9], and Ewing sarcoma (EYA3) [10]. The EYA tyrosine phosphatase activity promotes the repair of DNA damage [11,12] and could thus promote resistance to genotoxic cancer treatment measures. Furthermore there is evidence that the EYA tyrosine phosphatase promotes angiogenesis [13]. For these reasons inhibition of the EYA PTP is an attractive target for anti-cancer drug development. 

While PTPs have been sought-after drug targets for diseases ranging from obesity to cancer, success has traditionally been difficult. This has generally been attributed to the presence of a reactive active-site Cysteine that can confound high-throughput screens, the existence of over 100 PTPs with similar active-site stereo-chemistry making specificity challenging, and the fact that many identified PTP inhibitors tend to be charged mimetics of the substrate phospho-tyrosine. EYA has a unique advantage in this respect since it uses a mechanism that is different from that of the classical Cysteine-based PTPs; a nucleophilic Aspartate participates in a metal-dependent reaction similar to that carried out by the large family of haloacid dehalogenases [1,14].

In previous studies we reported that Benzbromarone (BBR), an anti-gout agent, could inhibit the EYA tyrosine phosphatase activity and was able to inhibit endothelial cell motility and angiogenesis [13]. BBR was a chronically administered anti-gout agent for over 30 years. However instances of hepatotoxicity caused it to be withdrawn from the US and some European markets in 2003 [15,16]. The toxicity has primarily been attributed to the metabolite 6-hydroxybenzbromarone (6OH-BBR) formed by the action of cytochromeP450 (specifically CYP2C9) [17,18]. Further sequential oxidation results in the catechol, 5,6-dihydroxybenzbromarone, and then a reactive ortho-quinone that could bind to cellular proteins via Cys residues [17]. In addition, BBR and derivatives compete with warfarin for CYP2C9 thus potentiating its anti-coagulant effect in patients receiving both drugs simultaneously [19]. Despite these concerns, the effectiveness of BBR as a uricosuric agent has kept its utility in the treatment of gout a subject of some debate [15].

The purpose of the present analysis was to determine whether known metabolites of BBR are EYA inhibitors and have anti-angiogenic activity, and to establish a structure-activity relationship for the inhibition of EYA phosphatase activity by compounds bearing the (1-benzofuran-3-yl) (4-hydroxyphenyl) methanone scaffold.

## Materials and Methods

### Ethics statement

All experiments were performed in accordance with institutional guidelines under Institutional Animal Care and Use Committee (IACUC) approval at Cincinnati Children's Hospital Research Foundation (CCHRF). IACUC at CCHRF approved the study described in this manuscript with Animal Use Protocol number 0D11086.

### Reagents

Human umbilical vein endothelial cells (HUVECs) were obtained from Lonza (Wakersville, MD USA) and maintained in Endothelial Growth Medium-2 (EGM-2) (Lonza, Walkersville, MD USA). Aortic ring assays were performed in Endothelial Basal Medium (EBM) obtained from Lonza (Walkersville, MD USA). WST-8 was obtained from Dojindo Molecular Technologies (Rockville, MD USA), puromycin and M199 from Life Technologies (Grand Island, NY USA). BBR and BZ were obtained from Sigma-Aldrich (St. Louis, MO USA) and stored as 10 mM stocks in DMSO (Sigma). VEGF165 was from R&D Systems (Minneapolis, MN USA), isolectin-B4 from Invitrogen Molecular Probes (Eugene, OR USA), fluorogel from Electron Microscopy Sciences (Hatfield, PA USA), and Matrigel from BD Biosciences (San Jose, CA USA). EYA3 antibody was obtained from Proteintech (Chicago, IL USA).

### Lentiviral infection of HUVECs

HUVECs were infected with lentivirus expressing shEYA3 (CCGGCCCTTCTACAAGTCCATCTTTCTCGAGAAAGATGGACTTGTAGAAGGGTTTTTG; NM_001990.2, Sigma-Aldrich, St Louis, MO) and selected with puromycin. Knock down of EYA3 was checked with Western Blot using EYA3 antibody.

### Enzyme assays

Purification of recombinant EYA3 has been reported previously [13]. Assays using the colorimetric substrate p-nitrophenylphosphate were conducted as previously reported [13]. 

### Proliferation assays

75% confluent HUVECs (<passage 8) were washed with PBS and trypsinized, deactivated with 10% FBS, spun down and counted. 1000 cells/well were seeded in triplicate in 100 μl EGM-2 complete medium either with inhibitors (in 0.2% DMSO) or vehicle alone (0.2% DMSO). After 0 hrs, 24 hrs, 48 hrs, 72 hrs of incubation at 37°C in 5% O_2_ 10 μl WST-8 was added to each well and incubated for 2 hours in the dark, at 37°C, 5% O_2_. Absorption was read at 450 nm. Fold change was calculated as the ratio of the OD_450_ at the experimental time point and at the start of the experiment (0 hours). 

### Wound healing assays

10^5^ HUVECs/well were seeded on 24-well plates and cultured until they reached confluency in EGM-2. The monolayers were then scratch-wounded using a 200 μl pipette tip, washed with PBS, and incubated with EGM-2 complete medium and either EYA Inhibitors or vehicle control (0.2% DMSO). Wounds were photographed immediately upon insult, and at various time-points following wounding using a phase-contrast microscope. The number of cells that migrated into the cleared area was counted using the Cell Counter function of ImageJ [20].

### Tubulogenesis assays

Growth factor reduced Matrigel was thawed overnight on ice at 4°C. 40 μl Matrigel was added to each well of a 96-well plate and incubated for 30 mins to polymerize. 10,000 cells in 100 μl EGM-2 complete medium with either inhibitors or vehicle control (0.2% DMSO) were added to each well. Plates were incubated for 24 hours at 37°C, 5% CO_2_ and then photographed at 5X magnification. Tube length was measured using the NeuronJ plugin [21] for the NIH ImageJ software [20].

### Aortic ring assays

The preparation of rat collagen and the aortic ring assay were carried out as previously described [22,23]. Briefly, aortic ring explants were obtained from C57BL/6 mice aged 4 to 6 weeks and cultured at 37°C in a humidified incubator under a 5% CO_2_ atmosphere in serum-free EBM. Within 30 minutes of dissection the rings were placed in a 3D collagen matrix supported by a UV-sterilized nylon mesh ring with inner and outer diameters of 3 mm and 5.6 mm, respectively. The matrix was prepared by mixing 8 parts of collagen solution, 1 part of 10x M199 and 1 part of 23.4mg/ml NaHCO_3_. After another 30 min incubation at 37°C, the assembly was transferred into a well of 96-well plate filled with 150 μl of culture medium; either control medium consisting of 0.1% DMSO in EBM, 2.5% FBS, penicillin/streptomycin and 20ng/ml VEGF165 or the test inhibitor dissolved in the control medium. Each compound was applied over a 10-day period with medium changes every two days. After culturing, explants were briefly washed in PBS and fixed (10 mins at room temperature) with 4% paraformaldehyde in PBS, washed and incubated in 2 μg/ml Isolectin-B4 for 3 hrs at room temperature. After three 10 min washes in PBS-Triton (PBS + 0.1% TritonX-100), the explants were mounted in Fluorogel and imaged with a laser microscopy scanning system (Zeiss, Goettingen, Germany). 

### Statistical analysis

Data are given as mean +/- SEM. Statistical analyses were performed using Graphpad Prism 5 for Mac OS X (Graphpad Software Inc., San Diego, CA USA). When three or more data sets were compared a one-way ANOVA with Dunnett’s post-test was used. When only two data sets were compared a Student’s t-test was used. P < 0.05 was considered statistically significant.

## Results

Synthesis of BBR metabolites and other derivatives has been previously reported [17]. Enzymatic assays were conducted using recombinant human EYA3 (a splice variant of Q99504 corresponding to isoform 2 and missing residues 1 - 126) purified as described [13]. The chromogenic substrate para-nitrophenylphosphate (pNPP) was used to measure catalytic activity of EYA3. The product of dephosphorylation, para-nitrophenol, is yellow under alkaline conditions hence its absorbance is measurable at 405nm. To assay potential EYA inhibitors, the compounds were pre-incubated with EYA3, pNPP added, and the formation of the p-nitrophenolate product measured. The previously characterized EYA inhibitors BBR and/or Benzarone (BZ) were used as positive controls. Since EYA3 has been shown to play a role in angiogenesis, the effect of the compounds on cell proliferation, migration and tube formation was assayed using primary human umbilical vein endothelial cells (HUVEC). In addition, an *ex vivo* examination of sprouting angiogenesis was conducted using the aortic ring assay [22].

### EYA3 inhibition

**Figure 1:**
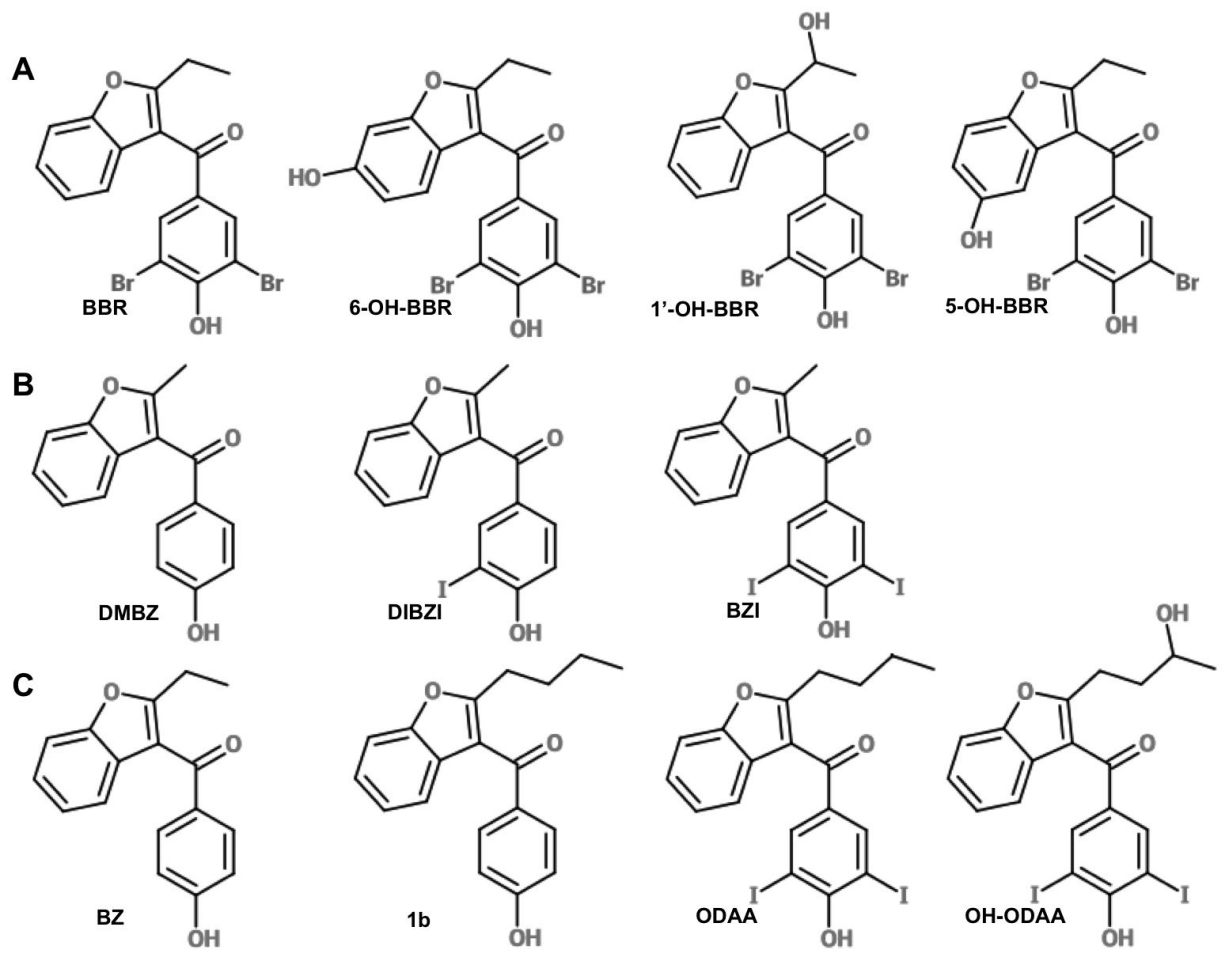
Chemical structures of compounds tested as EYA3 inhibitors and anti-angiogenic agents. (A) Metabolites of BBR. (B) Alternate halogenation of the phenol ring. (C) Alternate substituents at position 2 of the benzofuran.

The hydroxylated BBR metabolites 6OH-BBR and 1’-hydroxybenzbromarone (1’OH-BBR) (Figure 1A) are detected in plasma, bile and urine after oral administration [24]; 6OH-BBR is primarily the consequence of CYP2C9 – mediated metabolism, while 1’OH-BBR is the product of CYP3A4-mediated metabolism. Other proposed minor metabolites of BBR include 5OH-BBR (Figure 1A) and catechols derived from di-hydroxylation of the aryl ring of the benzofuran [17,18]. We assayed the EYA-inhibitory activity of synthetic 6OH-BBR, 5OH-BBR and 1’OH-BBR (Figure 1A and Table 1). 6OH-BBR had comparable *in vitro* EYA3-inhibitory potency to the previously described BBR derivative Benzarone (BZ), and had an IC50 over twice that of BBR. In contrast, 5OHBBR and 1’OH-BBR were significantly less effective EYA3 inhibitors.

**Table 1:** IC50 of BBR derivatives measured for pNPP hydrolysis by EYA3.

**Compound**	**IC50 μM** (95% confidence intervals)
BBR	8.1 (7.4 – 9.3) ^*a*^
6OH-BBR	21.5 (18.3 – 25.3)
1’OH-BBR	52.5 (33.9 – 81.5)
5OH-BBR	49.4 (23 – 105))
DMBZ	135.2 (121.9 – 150.1)
DIBZI	20.5 (18.9 – 22.2)
BZI	8.9 (8.5 – 9.4)
BZ	17.5 (16.2 – 18.8) ^*a*^
1b	9.7 (8.3 – 11.4) ^*a*^
ODAA	3.1 (2.6 – 3.7)
OH-ODAA	16.4 (14.9 – 17.9)

^a^ indicates IC50 values reported previously [13]

In previous studies we reported that the phenol moiety of BBR appears to be necessary for EYA binding and in docking studies the OH group forms a H-bond with Tyr329 of EYA3 [13]. We also noted that 3,5 dihalogenation of BBR yielded the highest binding affinities, with the dehalogenated form of BBR (BZ) having about half the affinity towards EYA3. These observations were consolidated using a related series of compounds: DMBZ (no halogen substituents and a 1-C alkyl side-chain at position 2), the mono-iodinated DIBZI, and the di-iodinated BZI (Figure 1B). Similarly the previously reported [13] compound 1b (4-C substituent at position 2, no halogenation) has a three-fold lower EYA3-binding affinity than the corresponding di-iodinated compound ODAA (Figure 1C). BZI is comparable in EYA3 inhibitory activity to BBR suggesting that the larger iodine atom can be accommodated in the predicted di-halo-phenol binding pocket. The dehalogenated compounds have lower lipophilicity and pKa values that are nearly three log units higher than their halogenated counterparts. Thus while BBR (pKa 4.5) is likely to exist as a phenolate ion at physiological pH [25], BZ and DMBZ are more likely to be uncharged. The increased affinities of the halogenated analogs are consistent with the phenol group binding within an anionic pocket of EYA3.

Two separate series of compounds allow us to assess the effect of alkyl-chain length at position 2 of the benzofuran ring: DMBZ, BZ, 1b, and BZI, ODAA, OH-ODAA (Figure 1B, C). In both cases increasing the length of the alkyl chain increases EYA3 inhibition,with ODAA being the most potent *in vitro* inhibitor assayed in this structure-activity relationship analysis. 

### Cell proliferation

**Figure 2:**
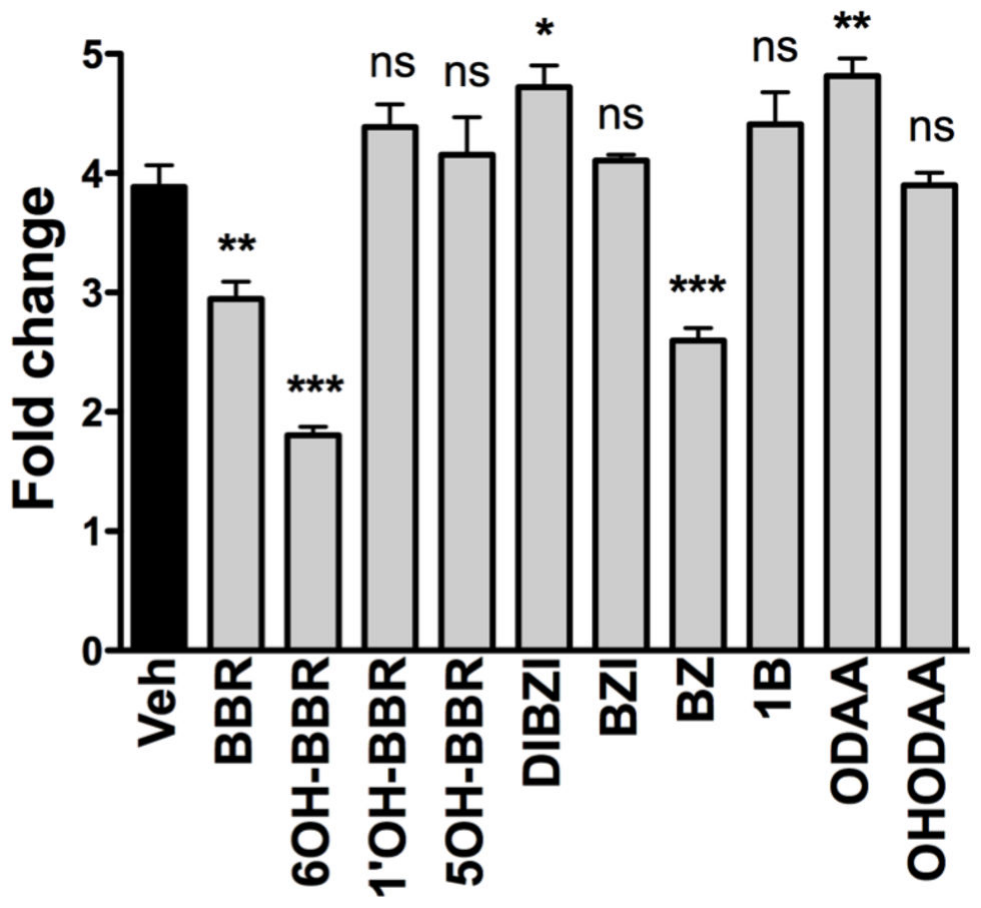
Proliferation of HUVECs in the presence of 7.5 μM test compounds. Cell viability was measured at 72 hours post-seeding. Asterisk represents significant differences between groups (*** P<0.001, * P<0.05).

A cell viability assay was conducted over 3 days of exposure to the test compounds in order to assess both the possibility of immediate cellular toxicity as well as any effect on cell proliferation. HUVECs were seeded and equilibrated for 24 hours before addition of the test compounds. Cellular metabolic activity was measured at defined time-points using the tetrazolium dye WST-8 to quantify NAD(P)H-dependent cellular oxidoreductase enzyme activity. 6OH-BBR showed over 50% reduction in cell proliferation. Treatment with BBR and BZ also reduced cell viability, but none of the other compounds tested had a negative impact on cell viability or proliferation (Figure 2). 

### Cell migration

**Figure 3:**
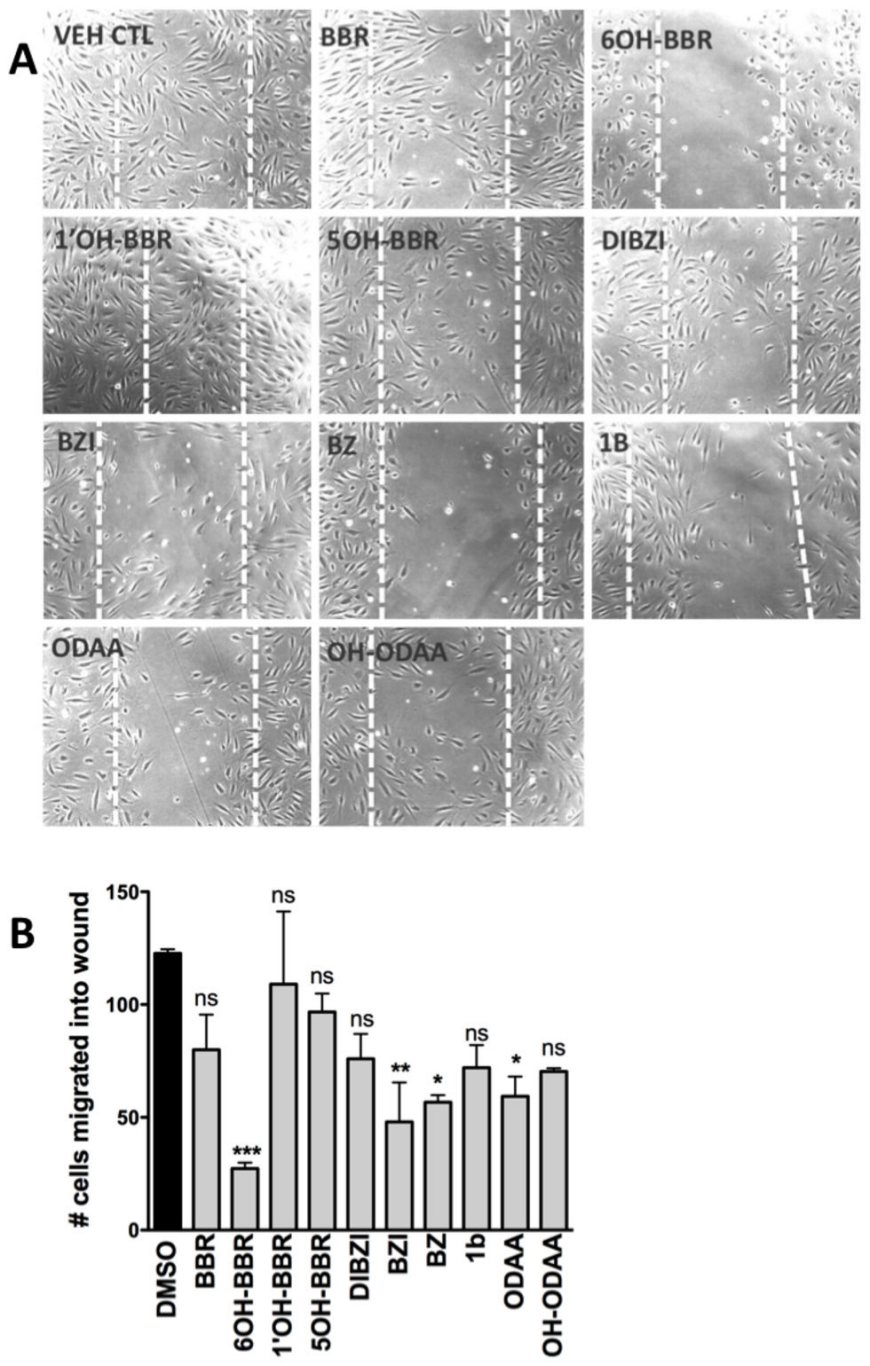
Lateral migration of HUVECs in the presence of test compounds. (A) HUVECs were scratch wounded and then cultured in the presence of either vehicle or 7.5 μM test compound. Representative phase-contrast photographs were taken 22 hours after wounding. White dotted lines indicate location of edges of the original wound. (B) Number of cells that migrate into the wounded area was counted using the Cell Counter functionality of ImageJ. Data represent three independent experiments and asterisk represent significant differences between groups (*** P<0.001, ** P<0.01, * P<0.05, ns not significant).

**Figure 4:**
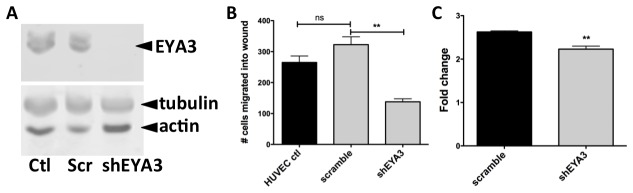
Effect of EYA3 knock-down on the migration and proliferation of HUVECs. (A) Western blot showing knockdown of EYA3 in HUVECs by infection with shEYA3 lentivirus relative to scramble (scr) non-specific RNA and control HUVEC cells. (B) EYA3 knock-down leads to a significant reduction in cell migration in a scratch wound healing assay. (C) EYA3 knock-down in HUVECs attenuates cell proliferation 72 hours post-seeding.

The effect of the test compounds on cell migration was assessed using a scratch wound healing assay and HUVEC cells. Cells were plated and grown to confluency. Scratch wounds were made using a sterile pipette tip, the medium changed to remove any cellular debris, and fresh medium with either vehicle or test compound was introduced. The number of cells that migrated into the cleared space was counted 22 hours later. As can be seen in Figures 3A and 3B, 6OH-BBR was the most effective inhibitor reducing cell migration by nearly 80%. Previous experiments had shown a role for EYA3 in trans-well migration of HUVECs [13]. These results were consolidated using wound healing assays on EYA3-knock-down HUVECs. Lentiviral shRNA targeting *EYA3* in HUVECs led to greater than 80% reduction in EYA3 protein levels (Figure 4A). Migration of EYA3-knock-down HUVECs was significantly reduced relative to control cells (Figure 4B). EYA knock-down also inhibited HUVEC proliferation (Figure 4C).

### Tubulogenesis

**Figure 5:**
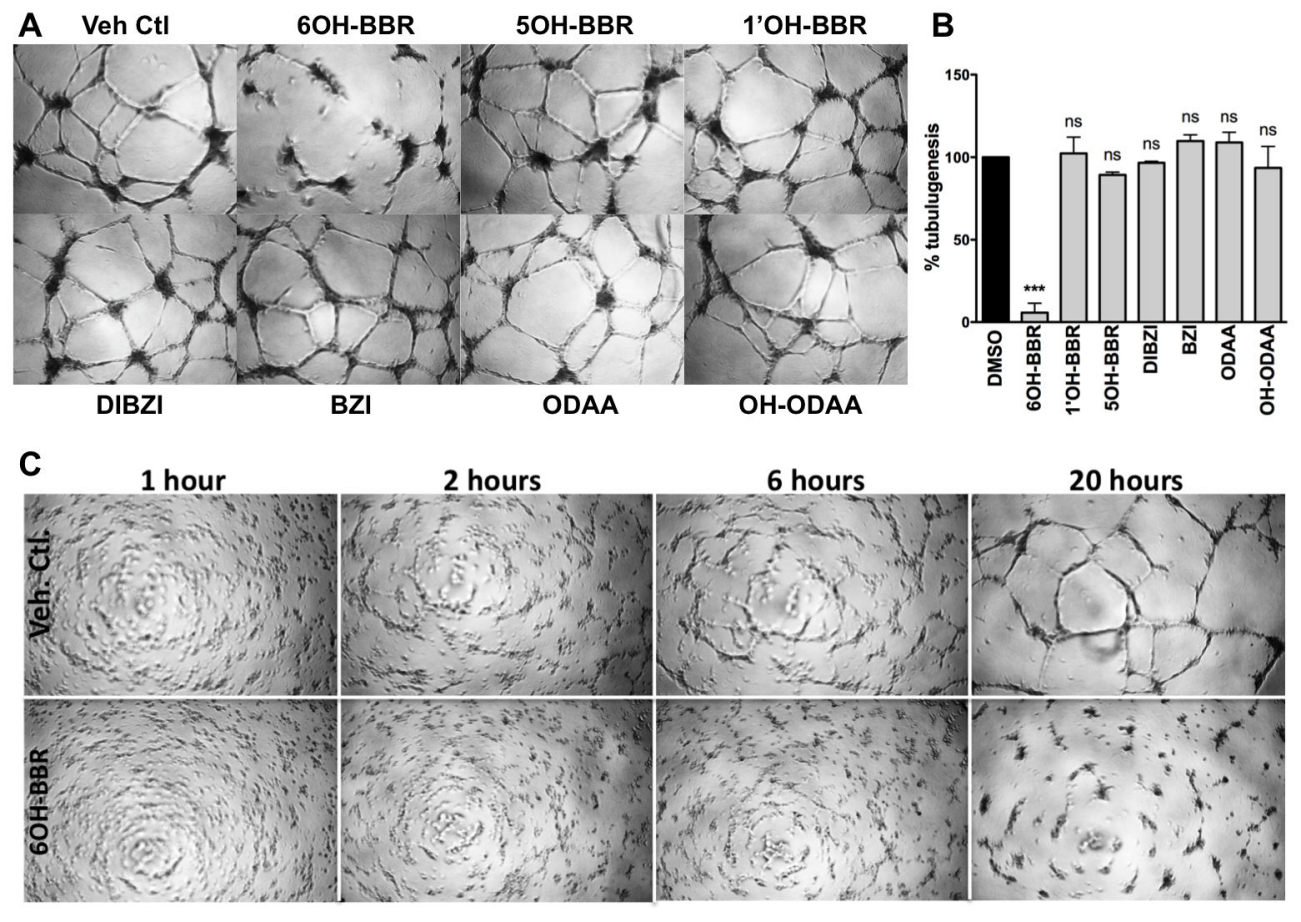
Effect of BBR derivatives on tubulogenesis of HUVECs. (A) Representative phase-contrast images of HUVECs plated on growth-factor reduced Matrigel in the presence of the indicated doses of test compounds, photographed 16 hours after plating. (B) Quantitation of the number of tube-like structures formed by HUVECs in the presence of test compounds. The number of tubes was measured using NeuronJ. Data are mean and standard error of two independent experiments. *p-values* from a one-way ANOVA are shown; ns is not significant, * P<0.05, ** P<0.01, *** P<0.001. (C) Time course of HUVEC tubulogenesis in the presence of either vehicle control (DMSO) or 6OH-BBR, photographed at 1 hour, 2 hours, 6 hours and 20 hours post-seeding.

Primary endothelial cells (ECs) on basement membrane rapidly attach, align themselves and form capillary-like tubules with lumens and tight cell-cell contacts. HUVECs seeded on Matrigel form relatively short tubes (no more than 2 – 3 ECs) and it has been suggested that these tubes represent the meeting of ECs that are pushing out or migrating from aggregates of ECs observed after a few hours of plating on Matrigel [26]. Previous studies have established a role for the EYA3 protein in cell migration [13,27] and tubulogenesis [13], and BBR and BZ were shown to inhibit the processes [13]. In the present study, 6OH-BBR had a much stronger inhibitory effect than BZ (Figure 5A, B). None of the other compounds tested showed significant attenuation of tube-formation. 6OH-BBR treated cells formed EC aggregates that did not migrate out and form tubes (Figure 5A). To better understand the stage in matrigel tube-formation affected by 6OH-BBR we followed cells plated on growth-factor reduced matrigel and treated with either vehicle or 6OH-BBR (Figure 5C). Differences become apparent within the first two hours and are clearly visible at the 6-hour time-point. The ECs are much less organized in the presence of 6OH-BBR. By 20 hours they form cellular aggregates but there are no tubules interconnecting the aggregates to form the characteristic microvascular network, supporting the suggestion that the inhibitor affects EC migration. The effect of 6OH-BBR (as well as BBR and BZ) on tube formation was attenuated in the presence of high concentrations of fetal bovine serum (FBS), likely reflecting non-specific protein binding.

### Aortic ring sprouting

**Figure 6:**
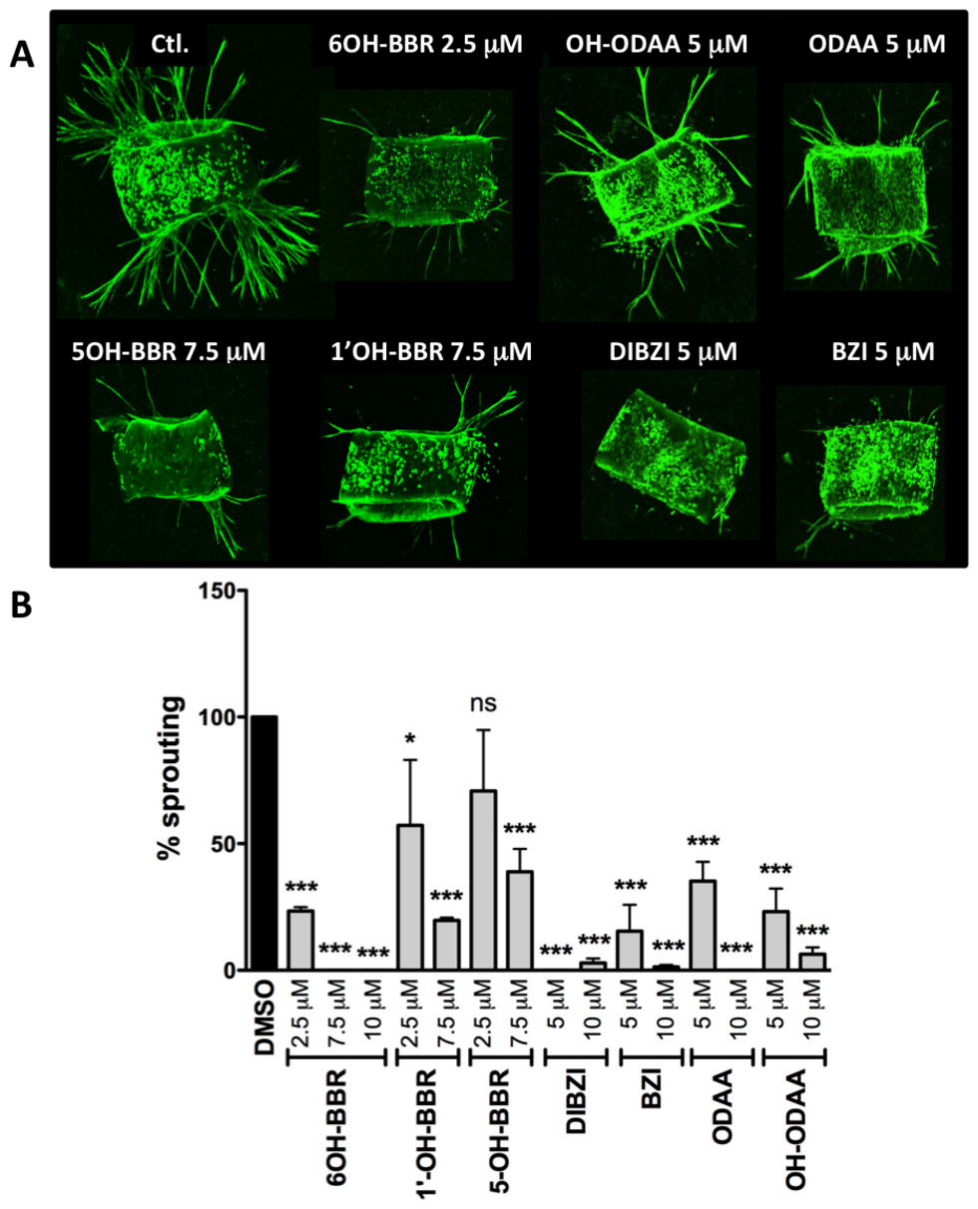
Effect of test compounds on aortic ring sprouting. (A) Representative images of isolectin-stained aortic rings treated with either vehicle controls or the indicated concentration of various test compounds. (B) Quantitation of the number of sprouts per ring with each bar representing at least 4 rings. *** P<0.001, ** P<0.01, * P<0.05, ns not significant).

This assay allows for analysis of cell proliferation, migration, tube formation, micro-vessel branching, and perivascular recruitment. When slices of mouse aorta are cultured in collagen gels there is typically a lag phase for the first 4 days followed by linear sprouting of endothelial cells. By day 6, branching is observed. All the test compounds were initially assayed at 7.5 μM and the number of branch-points counted. When there was a severe effect (no visible sprouting), or when there was no apparent effect, other doses were tested to establish a dose-dependence. All of the compounds assayed were able to attenuate aortic ring sprouting (Figure 6A, B). There was a strong reduction in initial sprouting and some attenuation of branching. With the exception of OH-ODAA, in all cases where there was sprouting, the length of sprouts was shorter than in the controls. These observations suggest that the effect of the compounds on angiogenesis was likely due to inhibition of endothelial cell migration. As in the previous assays 6OH-BBR was the most potent inhibitor. 

Aortic ring experiments are a physiologically relevant *ex vivo* model for angiogenesis since they monitor the development of lumenized blood vessels in the presence of supporting cell types such as macrophages and fibroblasts. However, for this same reason, the cellular targets for the inhibitory activity of 6OH-BBR cannot be unequivocally assigned.

## Discussion

Since BBR has been widely and successfully used in the treatment of gout for over 30 years there is a wealth of data on its safety, metabolism, pharmacokinetics, and both long-term and acute toxicity. The hepatotoxicity noted in its withdrawal from Western markets was a rare event (1:17,000) and there remains considerable debate regarding the risk versus benefits of BBR therapy [15,16]. Because of this history, BBR is an attractive candidate for drug re-purposing as well as being a lead compound for the development of more potent EYA inhibitors. The anti-angiogenic activity of EYA-inhibitory BBR derivatives could have potential utility in the treatment of cancer and other disorders of neovascularization such as the ocular conditions retinopathy of prematurity, diabetic retinopathy, and wet form age-related macular degeneration. EYA as a molecular target for the development of anti-angiogenic treatments would be distinct from the more commonly targeted VEGF-related pathways. Furthermore, because EYA3’s tyrosine phosphatase activity promotes DNA damage repair, EYA inhibitors could be used as neo-adjuvant therapy to improve the sensitivity of DNA damaging cancer treatments such as radiation and some forms of chemotherapy.

The original intention of this study was to determine whether hydroxylated metabolites or derivatives of BBR (6OH-BBR, 1’OH-BBR and 5OH-BBR) were able to inhibit EYA3. Previous pharmacokinetic studies had shown that BBR itself achieved a maximal plasma concentration of 3.9 μg/ml (~9.2 μM) in rapid eliminators and 9.17 μg/ml (~21.8 μM) in a slow eliminator, after a single oral dose of 100 mg [18], comparable to the EYA3-inhibitory IC50 value reported here for BBR. 6OH-BBR was comparable to the debromo-BBR analog Benzarone in its ability to inhibit the enzymatic activity of EYA3 *in vitro*, while 5OH-BBR and 1’OH-BBR were less inhibitory. 5OH-BZ (Benzarone (BZ) hydroxylated at the 5 position on the benzofuran ring) had IC50 > 150 μM, confirming that a polar substituent at this position disfavors EYA3 binding. Further structure-activity analyses revealed that halogenation of the phenyl ring and a longer alkyl side-chain at the 2 position of the benzofuran favored *in vitro* EYA3 inhibition. The most potent compound tested, in terms of inhibition of EYA3 catalytic activity, was ODAA, a metabolite of the anti-arrhythmia drug Amiodarone (AMIO). AMIO itself does not have the phenolic group necessary for EYA3 binding and is unlikely to be an EYA3 inhibitor.

The potential anti-angiogenic activity of BBR derivatives was assessed using three well-established cellular hallmarks of angiogenesis: proliferation, migration and tubulogenesis. In addition we used an *ex vivo* aortic ring sprouting assay to measure the effect of the EYA3-inhibitors in a more physiological context. Both cell migration and tubulogenesis were assayed within a 24-hour time-frame, while proliferation was monitored for three days. 6-OH-BBR was the only compound that attenuated cell proliferation at the 24-hour time-point. By 72 hours both BBR and BZ showed significant inhibition of cell proliferation. These results are consistent with our previous studies showing that BBR and BZ, and even reduction of EYA3 levels using shEYA3 does not influence proliferation of HUVECs in the first 24 hours [13]. Similarly, over-expression of EYA1 in the breast cancer cell line BT-474 shows an effect on longer-term cell proliferation (>3 days) while having little effect at 24 hours post-seeding [28]. This is relevant in light of the effects seen in relatively short-term experiments such as wound healing and tubulogenesis; the effects of BBR derivatives (except 6OH-BBR) on these cellular activities are likely not to be a secondary consequence of effects on cell proliferation. Interestingly several BBR derivatives (DIBZI, ODAA) seem to have a small but significant positive effect on cell proliferation.

6OH-BBR was the most potent inhibitor of cell migration, tube formation and aortic ring sprouting tested here. 6OH-BBR is a major metabolite of BBR and has a much longer half-life (30 hours relative to 3 hours for BBR as previously reported [24,29]) raising the interesting possibility that, in the context of angiogenesis, BBR may act as a pro-drug with biotransformation into 6OH-BBR yielding a pharmacologically active metabolite. It also suggests that polar substituents at position 6 of the benzofuran would yield more effective anti-angiogenic agents. In this context it is relevant that Benzbromarone was previously identified in a screen for PTP1B inhibitors [30]. Subsequent crystallographic analyses showed that BBR derivatives substituted at position 6 of the benzofuran ring bound at an allosteric site preventing closure of the catalytic WPD loop [31]. The mode of BBR derivative binding to PTP1B was significantly different from the docked conformations of BBR derivatives in EYA3; the phenol group of BBR lies along the PTP1B protein surface while it is inserted into a pocket in EYA3 with the OH group forming a hydrogen-bond with a tyrosine side-chain located on the floor of the pocket [13]. Commensurate with this, the phenolic OH in BBR appears to be necessary for EYA inhibition [13]. In contrast, PTP1B inhibition appears to be dependent on the benzofuran group since compounds lacking the dibromophenol moiety of BBR have been identified as PTP1B inhibitors [32].

Interestingly the most effective EYA3 inhibitor in enzymatic assays, ODAA, was a relatively weak inhibitor of tubulogenesis and cell migration. A likely explanation for this observation is high non-specific protein binding. Supporting this possibility is our previous observation that ODAA is highly bound to plasma protein (fraction bound ~99.9%) [33]. As a consequence, among the AMIO metabolites that can act as competitive inhibitors of CYP2C9-mediated warfarin metabolism and potentiate its anti-coagulant effect [34], ODAA was judged to be a minor contributor to the *in vivo* warfarin-AMIO drug-drug interaction [33].

In summary, the data reported here suggest that a major metabolite of BBR has potent anti-angiogenic activity in cellular and *ex vivo* assays. As a consequence the pharmacodynamics of BBR will be an important consideration in exploring the potential re-purposing of BBR as an anti-angiogenic agent. Further, the structure-activity relationships described provide insight into the design of more potent EYA inhibitors.
